# Chemical and perfusion markers as predictors of moyamoya disease progression and complication types

**DOI:** 10.1038/s41598-023-47984-y

**Published:** 2024-01-02

**Authors:** Jae Hyun Kim, Hanwool Jeon, Moinay Kim, Joonho Byun, Yeongu Chung, Si Un Lee, Wonhyoung Park, Jung Cheol Park, Jae Sung Ahn, Seungjoo Lee

**Affiliations:** 1 Department of Neurosurgery, Asan Medical Center, University of Ulsan College of Medicine, 88, Olympic-ro 43-gil, Songpa-gu, Seoul, 05505 Republic of Korea; 2 Department of Medical Science, Asan Medical Institute of Convergence Science and Technology, Asan Medical Center, University of Ulsan College of Medicine, Seoul, Republic of Korea; 3 Department of Neurosurgery, Korea University Guro Hospital, Seoul, Republic of Korea; 4 Department of Neurosurgery, Kangbuk Samsung Medical Hospital, Sungkyunkwan University School of Medicine, Seoul, Republic of Korea; 5 Department of Neurosurgery, Seoul National University Bundang Hospital, Seongnam, Republic of Korea; 6 Translational Biomedical Research Group, Asan Institute for Life Science, Asan Medical Center, Seoul, 05505 Republic of Korea; 7 Bio-Medical Institute of Technology, University of Ulsan College of Medicine, Seoul, Republic of Korea

**Keywords:** Health care, Medical research, Neurology, Signs and symptoms

## Abstract

To investigate the association between chemical markers (triglyceride, C-reactive protein (CRP), and inflammation markers) and perfusion markers (relative cerebral vascular reserve (rCVR)) with moyamoya disease progression and complication types. A total of 314 patients diagnosed with moyamoya disease were included. Triglyceride and CRP levels were assessed and categorized based on Korean guidelines for dyslipidemia and CDC/AHA guidelines, respectively. Perfusion markers were evaluated using Diamox SPECT. Cox proportional hazard analysis was performed to examine the relationship between these markers and disease progression, as well as complication types (ischemic stroke, hemorrhagic stroke, and rCVR deterioration). Elevated triglyceride levels (≥ 200) were significantly associated with higher likelihood of end-point events (HR: 2.292, CI 1.00–4.979, *P* = 0.03). Severe decreased rCVR findings on Diamox SPECT were also significantly associated with end-point events (HR: 3.431, CI 1.254–9.389, *P* = 0.02). Increased CRP levels and white blood cell (WBC) count were significantly associated with moyamoya disease progression. For hemorrhagic stroke, higher triglyceride levels were significantly associated with end-point events (HR: 5.180, CI 1.355–19.801, *P* = 0.02). For ischemic stroke, severe decreased rCVR findings on Diamox SPECT (HR: 5.939, CI 1.616–21.829, *P* < 0.01) and increased CRP levels (HR: 1.465, CI 1.009–2.127, *P* = 0.05) were significantly associated with end-point events. Elevated triglyceride, CRP, and inflammation markers, as well as decreased rCVR, are potential predictors of moyamoya disease progression and complication types. Further research is warranted to understand their role in disease pathophysiology and treatment strategies.

## Introduction

Moyamoya disease is characterized as a condition in which progressive stenosis and occlusion are observed in the terminal portion of the internal carotid artery (ICA), accompanied by the development of an abnormal vascular network around it^1^. These pathological features are not stagnant; they continuously cause vascular changes and progress, leading to ischemic and hemorrhagic strokes. The progression rate of moyamoya vessels is known to be approximately 20% in the non-surgical group^2^. Furthermore, the rebleeding risk in patients presenting with hemorrhage is 30–65%, and even asymptomatic patients have a 20–40% risk of stroke and progression. As a result of these findings, it is known that the prognosis can be improved through revascularization surgery in moyamoya disease patients with symptomatic strokes and hemodynamic compromise^1^. However, surgical, and medical treatments are not well-established for asymptomatic patients^3^, and there are cases where patients do not agree to surgical treatment or prefer medical treatment despite being candidates for surgery. Additionally, access to neurosurgeons capable of performing revascularization surgery may be limited, and some patients may have deteriorated general conditions or neurological states due to stroke, making surgical treatment difficult. In such cases, treatments are needed to delay the course of moyamoya disease and reduce the occurrence of additional strokes. However, research on risk factors for the progression of moyamoya disease is insufficient, and evidence for conservative treatment is not well-established.

Previously known progression factors included angiographic features (choroidal anastomosis, posterior cerebral artery involvement, collateral pathways, and vessel occlusion), decreased cerebral reserve capacity on single-photon emission computed tomography (SPECT), and prior stroke history^2,4–8^. However, many of these studies investigated progression risk factors by tracking both non-surgical and surgical groups, or focused on postoperative complications. Recent pathophysiological studies on moyamoya have revealed that metabolic factors, such as inflammatory disease, abnormal lipid metabolism, and RNF213-related genetic factors, play a significant role in the disease, but there is a lack of research on clinically meaningful blood biomarkers^9–13^. Therefore, we aim to investigate the risk factors for the progressive course of moyamoya disease by selecting only non-surgical patients and examining various factors, such as angiographic features, clinical history, blood biomarkers, and cerebrovascular hemodynamics, which may influence progression.

## Result

### Patient enrollment

**Figure 1:**
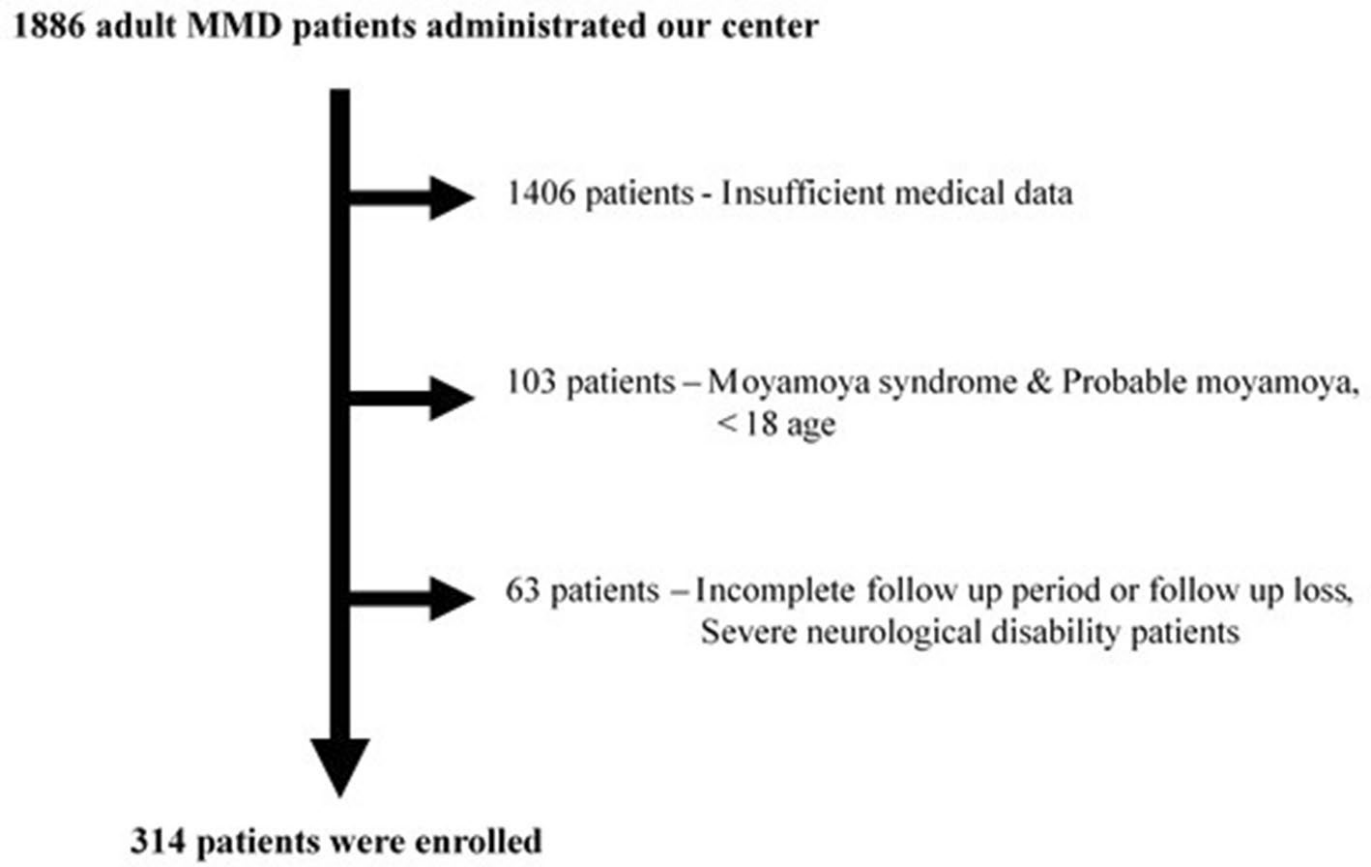
Patient enrollment flowchart. A flowchart showing the patient selection process for the study. Out of 1886 patients diagnosed with moyamoya disease at Asan Medical Center in Seoul between January 2005 and June 2022, 969 patients were excluded due to incomplete data, and 437 were excluded due to lack of TFCA or SPECT. Additionally, patients under 18 years old, those with moyamoya syndrome or probable moyamoya disease, and those with less than six months of follow-up or severe neurological deficits were excluded. Finally, 314 patients were included in the analysis.

Between January 2005 and June 2022, a total of 1886 patients were diagnosed with moyamoya disease at Asan Medical Center in Seoul. Of these, 969 patients were excluded due to incomplete blood sampling or insufficient clinical data. An additional 437 patients were excluded as they did not undergo TFCA or SPECT. Subsequently, patients under 18 years of age (24 cases), those with moyamoya syndrome (11 cases), and probable moyamoya disease (68 cases) were also excluded. Furthermore, patients with less than six months of follow-up (25 cases) and those with severe neurological deficits (> mRS score 4) (38 cases) were excluded as well. Consequently, a total of 314 patients were included in the final analysis (Fig. 1).

### Patient characteristics

The basal characteristics of the included cohort are summarized in Table 1. The mean age at first visit for the patients in the analysis was 42.5 ± 12.1 years (range 18–72), with a predominance of females (217, 69.1%). The mean follow-up duration was 38.2 ± 47.7 months. Among comorbidities, hypertension was the most prevalent at 39.5% (124/314), followed by diabetes at 16.6% (52/314). The mean body mass index (BMI) of the patients was 25.2 ± 4.4, which is considered high given that a BMI of 25 or higher is classified as obese in the Korean population^14^. Systolic blood pressure was 140.8 ± 21.0 mmHg, and diastolic blood pressure was 84.6 ± 14.0 mmHg. Both systolic and diastolic blood pressure values were above the American Heart Association (AHA) guideline for hypertension, suggesting a relatively high blood pressure trend among moyamoya patients^15^.

**Table 1:** Baseline characteristics of the moyamoya patients.

Variables	Value (%)
Mean follow up period (months)	38.42 ± 47.73
Sex: male	97 (30.9)
Age (year)	42.5 ± 12.1
Smoking	61 (19.4)
Alcohol	90 (28.7)
Hypertension	124 (39.5)
Diabetes mellitus	52 (16.6)
Dyslipidemia	82 (26.1)
Heart disease	26 (8.3)
MMD family history	33 (10.5)
Presenting	
Non-specific symptoms	59 (18.8)
Hemorrhage stroke	19 (6.1)
Ischemic stroke	236 (75.2)
Preoperative functional status	
mRS < 3	278 (88.5)
mRS ≥ 3	36 (11.5)
Suzuki stage	
< IV	238 (75.8)
≥ IV	76 (24.2)
rCVR findings on Diamox SPECT	
Normal	20 (6.4)
Mildly to moderately reduced	104 (33.1)
Moderately reduced	144 (45.9)
Moderately to severely reduced	46 (14.6)
Total cholesterol	165.5 ± 44.2
HDL	51.2 ± 0.8
LDL	102.4 ± 37.6
TG	119.9 ± 72.5
Albumin	4 ± 0.4
HbA1c	6 ± 1.2
Glucose	118.3 ± 46
Uric acid	4.7 ± 0.1
Creatinine	0.8 ± 0.2
CRP	0.3 ± 0.7
WBC	6.9 ± 2.1
Hb	13.4 ± 1.9
Homocysteine	13.6 ± 6.2
PLT	267.2 ± 70.6
Body mass index	25.2 ± 4.4
Height	162.1 ± 8.7
Weight	66.5 ± 14.4
Initial SBP	140.8 ± 21
Initial DBP	84.6 ± 14
PCA involvement	80(25.5)
Choroidal anastomosis	25(8.0)
Microbleed	146(46.5)

Data presented as mean ± SD (standard deviation) or percentage. MMD: moyamoya disease, mRS: modified Rankin Scale, rCVR: relative cerebrovascular Reserve, SPECT: single photon emission computed tomography, HDL: high-density lipoprotein, LDL: low-density lipoprotein, TG: triglyceride, HbA1C: hemoglobin A1C, CRP: c-reactive protein, WBC: white blood cell, Hb: hemoglobin, PLT: platelet, SBP: systolic blood pressure, DBP: diastolic blood pressure.

At the first visit, 255 patients presented with stroke as their presenting symptom, with ischemic stroke accounting for the majority (236, 75.2%), and hemorrhagic stroke cases being 19 (6.1%). Hemorrhagic stroke patients with an initial mRS score of 5 or higher were excluded from the study, leading to fewer cases enrolled. The most common presenting complaint was headache (59, 18.8%), followed by dizziness and seizures.

Brain perfusion SPECT analysis of relative cerebral vascular reserve (rCVR) revealed that 20 patients (6.4%) exhibited normal perfusion, 104 (33.1%) demonstrated mild impairment, 144 (45.9%) showed moderate impairment, and 46 (14.6%) had severe impairment. Thus, a reduction in rCVR was observed in the majority of patients (93.6%).

The Suzuki stage was analyzed with transfemoral cerebral angiography (TFCA). Most patients (278, 88.5%) had a score below 3, while 36 patients (11.5%) had a score of 3 or higher. In addition to the basic cell blood count, laboratory evaluations included the assessment of inflammation markers, such as white blood cell (WBC) count, C-reactive protein (CRP), and homocysteine levels. Cholesterol levels were also examined in relation to atherosclerotic changes.

### Association of relative cerebral vascular reserve (rCVR) and moyamoya disease progression

As previously defined, any newly developed cerebral stroke event, including hemorrhagic and ischemic events, and worsening of rCVR grade on SPECT during the follow-up period were considered events indicating Moyamoya disease progression. Out of the 314 patients, 72 (23%) experienced an event, i.e., a newly developed cerebral stroke or worsening of rCVR, during the follow-up period starting from their first visit. The average duration until the occurrence of an event signifying Moyamoya disease progression was 36.3 ± 13.6 months. Among the end-point events, ischemic stroke events occurred in 40 patients (13%), hemorrhagic stroke events in 20 patients (6.5%), and worsening of rCVR grade in 12 patients (4%). "The median follow-up time was 38.42 month. During a total of 1005 patient-years (12,061 months), 72 (22.9%) patients experienced end-point event. The total annual risk of end-point event occurrence during the follow-up period was approximately 7.2%/year, with an annual risk of 4.0%/year for ischemic events, 2.0%/year for hemorrhagic events, and 1.2%/year for worsening of rCVR grade. (Table 2)".

**Table 2:** Annual progression risk of each symptom.

	Angiographic progression event	Ischemic event	Hemorrhage event	worsening of rCVR
Annual risk of event	7.2%	4%	2%	1.2%

Cox proportional hazard analysis was employed to identify factors influencing end-point event occurrence. The analysis found that severe decreased rCVR findings on Diamox SPECT (HR: 3.906, CI 1.447–10.54, *P* = 0.01), elevated triglycerides (HR: 1.004, CI 1.000–1.007, *P* = 0.01), elevated white blood cell count (HR: 1.121, CI 1.017–1.235, *P* = 0.02), elevated CRP (HR: 1.392, CI 1.041–1.861, *P* = 0.03), and preoperative mRS ≥ 3 (HR: 2.076, CI 1.056–4.081, *P* = 0.03) were significant factors. The multivariate analysis included variables that had a p-value of < 0.05 in the univariate analysis. Backward elimination technique was used in the multivariate logistic regressions to assess the potential risk factors linked to the progression of moyamoya. Multivariate analysis revealed that severe decreased rCVR findings on Diamox SPECT (HR: 3.448, CI 1.269–9.368, *P* = 0.02), elevated triglycerides (HR: 1.004, CI 1.001–1.007, *P* = 0.01), and elevated CRP (HR: 1.399, CI 1.043–1.875, *P* = 0.03) emerged as significant independent factors (Table 3).

**Table 3:** Cox proportional hazards model indicating the predicting factors of progression in the moyamoya disease.

Variables	Univariable analysis		Multivariable analysis	
	Unadjusted HR	*p*-value	adjusted HR	*p*-value
Sex: male	0.521–1.487	0.633		
Age (year)	0.979–1.018	0.851		
Smoking	0.501–1.668	0.769		
Alcohol	0.628–1.795	0.824		
Hypertension	0.674–1.710	0.765		
Diabetes mellitus	0.478–1.731	0.773		
Dyslipidemia	0.978–2.550	0.062		
heart disease	0.741–3.417	0.076		
MMD family history presenting	0.147–1.109	0.079		
Non-specific symptoms	0.371–1.161	0.148		
hemorrhage stroke	0.500–3.087	0.64		
ischemic stroke	0.808–2.272	0.249		
Preop functional status				
mRS < 3				
mRS ≥ 3	1.056–4.081	**0.034**		
Suzuki stage				
< IV				
≥ IV	0.422–1.716	0.652		
rCVR findings on Diamox SPECT				
Normal			1	1
Mildly reduced	0.590–3.435	0.432	0.496–2.939	0.679
Moderately reduced	0.562–3.368	0.486	0.505–3.055	0.638
Severely reduced	1.447–10.540	**0.007**	1.269–9.368	**0.015**
Total cholesterol	0.990–1.001	0.099		
HDL	0.976–1.009	0.356		
LDL	0.991–1.004	0.481		
TG	1.001–1.007	**0.013**	1001–1.007	**0.014**
Albumin	0.417–1.357	0.344		
HbA1c	0.876–1.296	0.527		
Glucose	0.999–1.009	0.107		
Uric acid	0.759–1.070	0.234		
Creatinine	0.220–1.922	0.436		
CRP	1.041–1.861	**0.025**	1.043–1.875	**0.025**
WBC	1.017–1.235	**0.021**		
Hb	0.816–1.061	0.282		
Homocysteine	0.922–1.037	0.452		
PLT	0.998–1.005	0.454		
Body mass index	0.945–1.058	0.997		
Height	0.950–1.006	0.129		
Weight	0.977–1.013	0.55		
Initial SBP	0.995–1.018	0.302		
Initial DBP	0.995–1.030	0.165		
PCA involvement	0.375–1.524	0.435		
Choroidal anastomosis	0.767–3.351	0.210		
Microbleed	0.650–1.662	0.871		

MMD: moyamoya disease, mRS: modified Rankin Scale, rCVR: relative cerebrovascular Reserve, SPECT: single photon emission computed tomography, HDL: high-density lipoprotein, LDL: low-density lipoprotein, TG: triglyceride, HbA1C: hemoglobin A1C, CRP: c-reactive protein, WBC: white blood cell, Hb: hemoglobin, PLT: platelet, SBP: systolic blood pressure, DBP: diastolic blood pressure, PCA: posterior cerebral artery.

Significant values are in bold.

These findings confirmed that factors causing inflammatory changes in blood vessels, such as triglycerides, C-reactive protein (CRP), and white blood cell (WBC) count, can influence the progression of Moyamoya disease. Moreover, in line with previous studies, it was determined that decreased relative cerebral vascular reserve (rCVR) can affect the prognosis of patients with this condition.

### Chemical markers predicting moyamoya disease progression: triglyceride & CRP and inflammation marker

A total of 314 patients diagnosed with moyamoya disease were included in this study. The study examined the association between chemical markers, such as triglyceride and C-reactive protein (CRP), and the progression of moyamoya disease.

**Figure 2:**
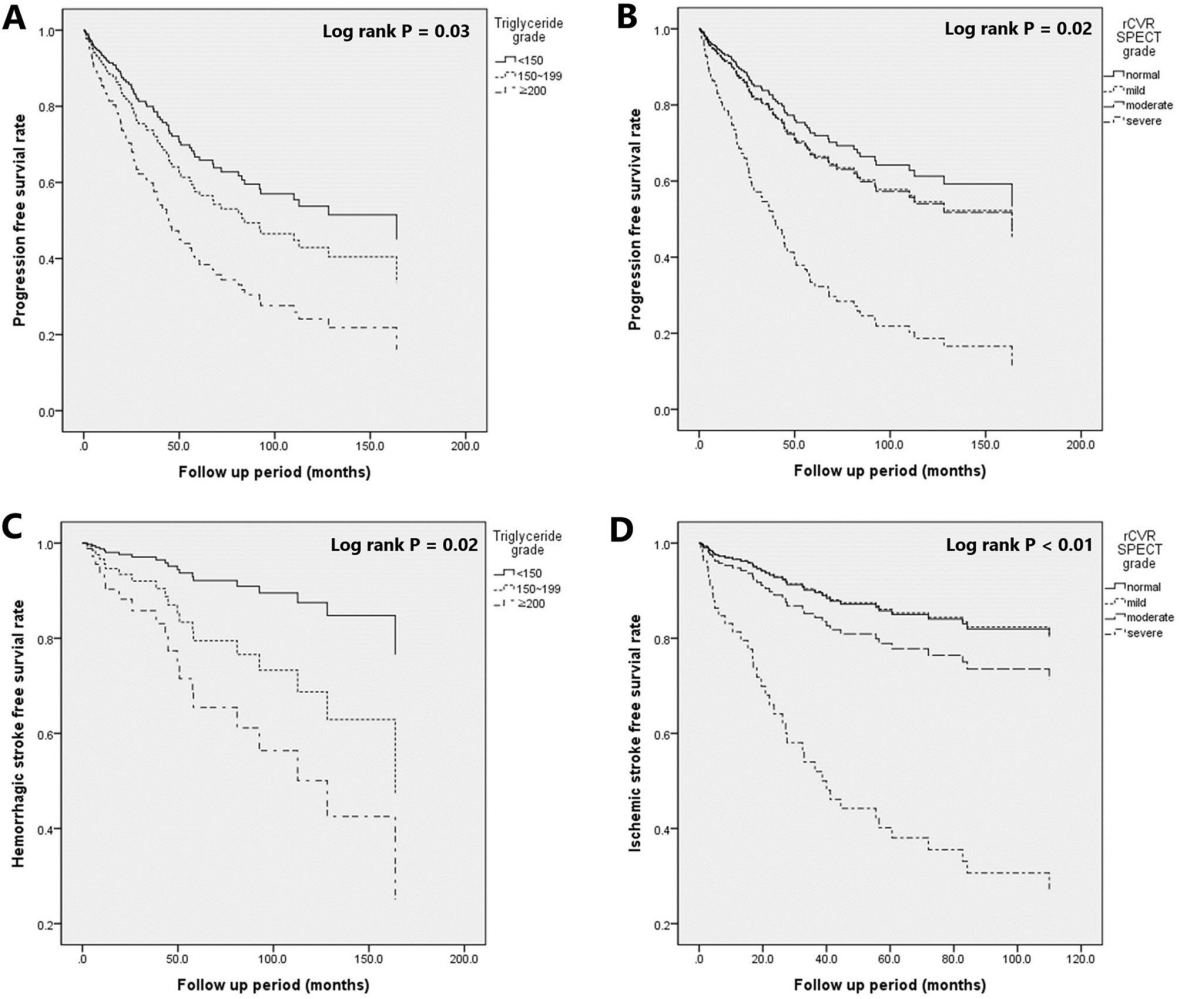
Progression free survival depending on serum triglyceride level and relative cerebrovascular reserve (rCVR). This study aimed to investigate the relationship between chemical markers and the progression of moyamoya disease. The study found that patients with triglyceride levels of 200 or higher had a significantly higher likelihood of experiencing end-point events compared to those with triglyceride levels of less than 150 (HR: 2.292, CI 1.00–4.979, *p* = 0.03) (**A**). The Kaplan–Meier cumulative curves illustrate that patients with severe decreases in Diamox SPECT grade had a significantly higher likelihood of experiencing end-point events compared to those with normal grade SPECT (HR: 3.431, CI 1.254–9.389, *p* = 0.02) (**B**). For hemorrhagic stroke, the study found that patients with triglyceride levels of 200 or higher had a significantly higher likelihood of experiencing hemorrhagic stroke compared to those with triglyceride levels of less than 150 (HR: 5.180, CI 1.355–19.801, *p* = 0.02) (**C**). Patients with severely decreased rCVR as assessed by Diamox SPECT demonstrated a significantly higher risk of experiencing ischemic stroke events compared to those with normal SPECT findings (hazard ratio [HR]: 5.939, 95% confidence interval [CI]: 1.616–21.829, *P* < 0.01) (**D**).

Triglyceride levels were categorized according to the Korean guidelines for the management of dyslipidemia. The study found that patients with triglyceride levels of 200 or higher had a significantly higher likelihood of experiencing end-point events, which means any type of clinical stroke and decreased cerebrovascular reserve compared to those with triglyceride levels of less than 150 (HR: 2.292, CI 1.00–4.979, *P* = 0.03) (Fig. 2A). The Kaplan–Meier cumulative curves showed that patients with severe decreased grade on Diamox SPECT had a significantly higher likelihood of experiencing end point events compared to those with normal grade SPECT (HR: 3.431, CI 1.254–9.389, *P* = 0.02) (Fig. 2B).

Additionally, the study examined the association between CRP levels, an inflammation marker, and the progression of moyamoya disease. The mean level of CRP among the study participants was 0.3, with 6 cases demonstrating levels above 1.0 mg/L. According to the Centers for Disease Control (CDC) and the American Heart Association, CRP levels are generally categorized as low risk (< 1.0 mg/L), average risk (1–3 mg/L), or high risk (> 3 mg/L). Although no significant subgroups could be categorized, the observed increase in CRP levels suggested a potential association between inflammatory markers and the progression of moyamoya disease. Furthermore, a one-sided t-test showed that an increase in white blood cell (WBC) count was also significantly associated with the progression of moyamoya disease.

Overall, these findings provide insights into the potential role of chemical markers, such as triglyceride and CRP, in predicting the progression of moyamoya disease. The observed association between inflammatory markers and moyamoya disease warrants further investigation and may inform future studies aimed at understanding the pathophysiology of the disease and developing effective treatment strategies.

### Type of complication of moyamoya disease and chemical and perfusion markers

We also aimed to examine the association between chemical and perfusion markers and the type of complication in moyamoya disease (Table 4). The study divided the end point events into three subgroups: ischemic stroke, hemorrhagic stroke, and deterioration of rCVR. Cox proportional hazard analysis was performed for each subgroup, and the results were compared with previously identified risk factors.

**Table 4:** Multivariate analysis of each progression event with CRP, TG, rCVR grade on SPECT.

	Multivariate	adjusted HR	P value
Ischemic event			
TG < 150	1		
TG 150 ~ 199	0.938	0.382–2.301	0.888
TG > 200	1.209	0.357–4.102	0.760
rCVR findings on Diamox SPECT			
Normal	1		
Mildly reduced	0.975	0.270–3.521	0.969
Moderately reduced	1.544	0.441–5.400	0.497
Severely reduced	5.939	1.616–21.829	**0.007**
CRP	1.465	1.009–2.127	**0.045**
Hemorrhagic event			
TG < 150	1		
TG 150 ~ 199	2.808	0.923–8.549	0.069
TG > 200	5.180	1.355–19.801	**0.016**
rCVR findings on Diamox SPECT			
Normal	1		
Mildly reduced	0.875	0.176–4.361	0.875
Moderately reduced	1.193	0.244–5.835	1.193
Severely reduced	0.766	0.062–9.398	0.766
CRP	1.636	0.979–2.733	0.060
Worsening of rCVR grade on SPECT			
TG < 150	1		
TG 150 ~ 199	0.660	0.083–5.215	0.693
TG > 200	3.657	0.781–17.123	0.100
CRP	0.394	0.015–10.187	0.575

CRP: c-reactive protein, rCVR: relative cerebrovascular Reserve, SPECT: single photon emission computed tomography, TG: triglyceride.

Significant values are in bold.

For hemorrhagic stroke, the study found that patients with triglyceride levels of 200 or higher had a significantly higher likelihood of experiencing end point events compared to those with triglyceride levels of less than 150 (HR: 5.180, CI 1.355–19.801, *P* = 0.02) (Fig. 2C). However, no significant association was found between CRP or rCVR deterioration and hemorrhagic stroke.

For ischemic stroke, the study found that patients with severe decreased rCVR findings on Diamox SPECT had a significantly higher likelihood of experiencing end point events compared to those with normal grade SPECT (HR: 5.939, CI 1.616–21.829, *P* < 0.01) (Fig. 2D). Additionally, an increase in CRP levels was found to be significantly associated with the occurrence of ischemic stroke (HR: 1.465, CI 1.009–2.127, *P* = 0.05). However, no significant association was found between worsening of rCVR grade and ischemic stroke.

Overall, these findings suggest that chemical and perfusion markers may be useful in predicting the type of complication in moyamoya disease. The observed association between triglyceride levels and hemorrhagic stroke, and severe decreased rCVR findings on Diamox SPECT and ischemic stroke, warrants further investigation and may inform future studies aimed at understanding the pathophysiology of moyamoya disease and developing effective treatment strategies.

## Discussion

Moyamoya disease is a progressive disorder that causes occlusive changes in the internal carotid artery, increasing the probability of subsequent ischemic stroke^2,5^. In addition, the formation of moyamoya vessels and collateral pathways around the stenotic blood vessels increases the risk of intracranial hemorrhage due to the fragility of these vessels^16^. Therefore, revascularization surgery is recommended for patients with hemorrhagic or ischemic events and hemodynamically compromised patients, as it has been shown to improve long-term clinical outcomes and reduce the risk of recurrent stroke^1^. However, treatment options for asymptomatic moyamoya patients remain undefined, and conservative treatment has been associated with a 10% risk of stroke events and a pathological progression^2^. The AMORE study in Japan is currently investigating treatment guidelines for asymptomatic moyamoya patients^3^.

Previous research on the natural course of moyamoya disease by Kuroda et al. showed that approximately 20% of patients exhibited progression of occlusive lesions on angiography, regardless of the presence or absence of symptoms^2^. The study identified female sex as a significant risk factor. In another study of 40 asymptomatic moyamoya patients, seven symptomatic stroke events were observed, and disturbed hemodynamics were identified as a risk factor^4^. However, these studies may have been influenced by the inclusion of patients who underwent surgery or the contralateral hemisphere in patients who did not undergo surgery. Our study aimed to investigate the natural course of moyamoya disease more accurately by examining only patients who did not undergo surgery. In our cohort of 314 patients, clinical progression was observed in 72 (23%) patients, and the annual any event risk ratio was 7.2%.

Moyamoya disease is a progressive disorder with changes in angiographic features and the brain's hemodynamic reserve state. The term "progression" has been used in various contexts in the literature, referring to stroke events, hemodynamic changes, or angiographic changes such as ICA stenosis, moyamoya vessel formation, and collateral pathway development. Angiographic progression has been reported in patients with serial angiography, showing occlusive progression in the ICA and identifying female sex and blood pressure as risk factors^2,4^. Studies on stroke event occurrence have identified thyroid disease, previous stroke history, microbleeds, anterior choroidal artery anastomosis, and advanced Suzuki state as risk factors^7,8,17–19^. Additionally, decreased cerebrovascular reserve has been reported to increase the risk of ischemic stroke^5,16,17^. Although angiographic occlusive changes were previously considered the progression of moyamoya disease, recent recommendations suggest including brain reserve capacity in determining progression and prognosis due to the limited information provided by angiographic features^16^. In our study, we defined progression as any stroke event or reduced hemodynamic reserve and investigated risk factors for these events. We identified high triglycerides, elevated C-reactive protein (CRP), and decreased brain reserve in SPECT as independent risk factors.

Multiple studies have suggested that the progression of Moyamoya disease is associated with inflammatory changes^20^. An increase in pro-inflammatory cytokines, such as IL6, IL-17, IL23, and TNFα, has been observed in blood tests performed on Moyamoya patients^22–23^. The increase in these pro-inflammatory cytokines is believed to activate RNF213 transcription, which subsequently triggers intima thickening and angiogenesis development through various pathways, ultimately leading to the onset of Moyamoya disease^9,20^. Furthermore, the increase in pro-inflammatory cytokines induces an elevation in CRP by binding to hepatocyte CRP transcription factors in the liver. This increase in CRP has been linked to atherosclerotic development and plasma cholesterol levels, as well as a proportionate increase in pro-inflammatory cytokines and serum and CSF levels in studies examining the role of sortilin, an intracellular protein, in Moyamoya disease^23^. Elevated CRP levels have also been associated with poor prognosis in atherosclerotic coronary disease^24^. Our study confirmed that elevated CRP levels are a progressive risk factor in Moyamoya patients. However, further research is needed to investigate the direct mechanism connecting CRP levels and Moyamoya progression.

Our study found a correlation between the progression of Moyamoya disease and high triglycerides (TG). Previous studies have reported an increased prevalence of hyperlipidemia in Moyamoya patients (27.7–37.3% vs. 16.3%)^13,25^. However, the specific type of cholesterol associated with the progression of Moyamoya disease has not been thoroughly investigated. Our study identified that neutral lipids were an independent risk factor for the progression of Moyamoya disease. Neutral lipids can damage endothelial cells and smooth muscle cells in blood vessels, increase oxidative stress, form foam cells, and impair the vascular healing process^26,27^. Additionally, TG-rich lipoproteins have been shown to promote monocyte recruitment, attachment, and plaque formation, ultimately leading to atherosclerotic changes^28,29^. Previous Moyamoya disease research has rarely found evidence of lipid deposition compared to evidence of smooth muscle cell hyperplasia and fibrocellular thickening. However, recent studies suggest that RNF213 mutations may affect lipid droplet targeting and impair fat-stabilizing activity, leading to a focus on lipid metabolism as a key factor in Moyamoya disease development^12,30^. In this study, triglyceride was also shown as an independent risk factor for progressive moyamoya, and the average BMI of moyamoya patients was 25, which is higher than the normal Korean average^14^. This indirectly demonstrates the association of lipid metabolism with the progression of moyamoya disease.

Considering the potential benefits of predicting MMD progression, it is necessary to develop clinically applicable methods for predicting Moyamoya disease. Current clinical practice evaluates and predicts the progression of Moyamoya disease using SPECT instead of blood markers. Patients with decreased rCVR are at a higher risk for ischemic stroke events. This information can be used to distinguish between asymptomatic patients and those with decreased rCVR, who may require more aggressive treatment. In this study, we confirmed the association of Moyamoya disease progression with inflammatory changes and lipid metabolism disorders by observing hemodynamic changes through SPECT, as well as increased CRP and triglyceride levels. No protocols or studies have been conducted regarding anti-inflammatory management or lipid profile medication in MMD treatment. However, as increasing evidence links inflammatory markers and lipid profile increases to Moyamoya disease progression, further research is needed to determine whether medical control of these factors can improve prognosis in Moyamoya disease progression. If confirmed, these modifiable factors could lead to treatments that alleviate the worsening of Moyamoya disease through lifestyle improvements, exercise, and medication.

Our study has several limitations. Firstly, there may be referral bias from tertiary care institutions, as the study population was drawn from such centers. Secondly, our study design was a retrospective cohort study; however, the large sample size helps to mitigate this limitation.

In the case of symptomatic patients, the natural course of the disease may be unclear due to surgical treatments censoring the patients' disease progression. However, our study aimed to investigate the pre-surgical natural course of the disease, making it a meaningful contribution to the field.

## Conclusion

In conclusion, our study has demonstrated that elevated CRP levels and high triglycerides are independently associated with the progression of Moyamoya disease. These findings contribute to the growing body of evidence suggesting that inflammation and lipid metabolism disorders are implicated in the pathophysiology of Moyamoya disease. Despite the limitations of our study, such as referral bias and the retrospective design, our findings provide valuable insights into the pre-surgical natural course of the disease. Further research is needed to explore the potential benefits of anti-inflammatory and lipid-lowering interventions in the management of Moyamoya disease. Ultimately, understanding and addressing modifiable factors, such as inflammation and lipid metabolism, may lead to improved therapeutic strategies and outcomes for patients with Moyamoya disease.

## Methods

### Disease definition

Moyamoya disease is defined as a condition in which progressive stenosis and occlusion are observed in the terminal portion of the internal carotid artery (ICA), accompanied by the development of an abnormal vascular network (moyamoya blood vessels) around it^1^. These vascular features should be diagnosed using MR head angiography (3.0 Tesla), or transfemoral cerebral angiography (TFCA). Moyamoya disease is limited to cases caused by unknown etiology, without any underlying factors. For example, a history of atherosclerosis, autoimmune disease, meningitis, brain tumors, Down's syndrome, von Recklinghausen's disease, traumatic brain injury, or brain radiation must not be present to define it as moyamoya disease^1^.

Moyamoya syndrome is defined as a condition where there is a history of factors that can trigger the development of an abnormal vascular network, along with the presence of distal internal carotid artery (ICA) stenosis^31^. Additionally, probable moyamoya disease refers to the cases where the cause is unclear, but stenosis or occlusion and moyamoya vessel features are observed in unilateral distal ICA^31^.

## Population and method

### Study population

The study included patients diagnosed with moyamoya disease who visited Seoul Asan Hospital from January 2005 to June 2022. This study was designed as the prospective observational study recruited in single medical center. The diagnosis of moyamoya disease was based on the diagnosis criteria (2012 guidelines for diagnosis and treatment of moyamoya disease, the Research Committee on Moyamoya Disease in Japan)^1^. Only patients with moyamoya disease confirmed through transfemoral cerebral angiography (TFCA) were enrolled in this study.

The inclusion criteria for patient enrollment in this study were: (1) adults aged 18 or older, (2) patients who were followed up for at least six months. However, during the follow-up period, even if it was less than six months, patients who experienced an endpoint event or underwent revascularization surgery were included in the patient group, with the follow-up period lasting until the corresponding day. (3) Only patients with a modified Rankin Scale (mRS) score of 4 or lower at the time of their visit were targeted.

Patients with a history of factors that could cause secondary moyamoya syndrome, such as atherosclerosis, autoimmune disease, meningitis, brain tumors, Down's syndrome, von Recklinghausen's disease, head injury, and radiation treatment in the brain, were excluded from this study. Additionally, patients with probable moyamoya, such as unilateral moyamoya disease (MMD) or moyamoya syndrome (MMS), were also excluded.

### Data collection

All methods in this study related to patient were approved by institutional review board (IRB) of Asan Medical Center. (IRB No.2022-0685).

Baseline clinical characteristics, radiologic and laboratory data, and outcomes were collected retrospectively by blinded research nurses and physicians. The analysis was performed using data obtained at the patient's first admission. Clinical characteristics included age, height, weight, body mass index (weight (kg)/height(m^2^)), smoking history(for more than one year), alcohol use, hypertension (systolic blood pressure ≥ 140 mmHg at presentation and history of taking antihypertensive medications), history of diabetes mellitus (history of taking diabetic medications and fasting blood glucose ≥ 126 mg/dl), heart disease (previous history of ischemic heart disease or taking related medications), family history of moyamoya disease, and initial presenting symptoms. Neurological status at presentation was assessed using the modified Rankin Scale (mRS) score. When patients presented with symptoms such as headache, dizziness, or seizures, they were classified as "asymptomatic."

A "hemorrhagic event" was noted when evidence of hemorrhage was evident on brain computed tomography. Ischemic presentation was defined when ischemic infarction was confirmed on brain CT and MR (diffusion weighted image and FLAIR) or neurological symptoms occurred due to transient ischemic attack. Laboratory data collection was performed using fasting blood samples taken in the morning after presentation, and the following parameters were evaluated: total cholesterol, high-density lipoprotein (HDL) cholesterol, low-density lipoprotein (LDL) cholesterol, triglyceride (TG), albumin, HbA1c, glucose, uric acid, creatinine, C-reactive protein (CRP), white blood cell count, hemoglobin, platelet count, and homocysteine.

To assess dynamic cerebrovascular insufficiency using SPECT, patients undergo both basal and acetazolamide-stress brain perfusion SPECT. The "10-bend" color palette technique is utilized to represent the SPECT image grades, with cerebellar perfusion set as the reference. Relative cerebrovascular reserve (rCVR) is evaluated by comparing it to the cerebellar blood volume.

Abnormal cerebral perfusion is considered when showing abnormal cerebral perfusion below 60% of cerebellar perfusion. Reduced regional cerebrovascular reserve is defined as a decrease in perfusion in at least one-third of the ACA or MCA territories compared to basal SPECT after acetazolamide administration^32^. The perfusion decrease is evaluated using digitalized color change. A "mild decrease in reserve" is defined as a change in one color spectrum, indicating a decrease in rCVR by more than 10% compared to basal SPECT. A "moderate decrease in reserve" is defined as a change in two or more color spectrums, indicating a decrease in rCVR by more than 20%. A "severe decrease in reserve" is defined as the presence of a steal phenomenon in addition to the changes observed in "moderate decrease in reserve."

SPECT results demonstrating these hemodynamic changes are evaluated by experienced nuclear medicine physicians to determine the stage of moyamoya disease. In cases of both cerebral hemispheres showed different rCVRs, the more advanced grade was adopted.

Suzuki stages were divided into 1 to 6 based on the TFCA findings, and in case of both sides showed different grades, the higher grade was adopted by two experienced radiologic physicians. Additionally, we classified dichotomously patients into two groups: early collateral moyamoya state group (Suzuki stage 1–3) and late collateral moyamoya state group (Suzuki stage 4–6)^33^. Posterior cerebral artery (PCA) involvement was defined as the occurrence of stenosis or occlusion of 50% or more in the P1 to P3 segment of either PCA. The definition of choroidal anastomosis involves the connection of the choroidal artery with the medial end of the medullary artery. Microbleed was defined as the presence of areas of small, circular hypointense changes on T2-weighted MRI.

### End-point (event) definition

We set the end point as any newly developed cerebral stroke event, including hemorrhagic and ischemic events, and worsening of rCVR grade on SPECT during the follow-up period after the first visit. Ischemic stroke was defined as cases with neurological symptoms manifested or newly developed cerebral infarctions confirmed on MRI, including transient ischemic attacks. Hemorrhagic stroke was defined as cases diagnosed with newly developed hemorrhage evidence on brain CT. An event was considered as a one-grade or higher deterioration in rCVR observed in the follow-up SPECT.

The reason for setting up the end-points was to reflect the indications of moyamoya surgical treatment guidelines, as it represents a condition that requires additional surgical treatment. The reason for including SPECT rCVR grade as one of the end-points is as follows: the decrease in rCRV itself is included in moyamoya surgical indications, and as emphasized in the Berlin grading system and additional validation studies, the decrease in rCVR significantly affects the prognosis of patients. During the follow-up period, if the patient met the surgical indications in the moyamoya surgical treatment guidelines and agreed to surgery, the operation was performed, and the time of surgery was set as the censored date.

### Clinical follow-up

Follow-up observations were conducted through outpatient visits every six months. After the first year following the initial visit, MRA was performed to evaluate vasculature changes, and then every two years thereafter. Patients were also recommended to visit the outpatient clinic or emergency room in case of neurological symptom occurrence. The follow-up was primarily based on outpatient visits, and for those who had difficulty visiting, a telephone survey was conducted. It was recommended to perform brain perfusion SPECT every two years after the first examination.

Patients who experienced hemorrhage symptoms were actively managed with blood pressure control by taking blood pressure medication to ensure their SBP did not exceed 140 mmHg, and they did not take aspirin or antiplatelet agents. Patients who visited with ischemic symptoms were monitored with taking aspirin, a single antiplatelet agent. Asymptomatic patients did not take an additional antiplatelet medication due to concerns about hemorrhage symptom occurrence. All patients included in this study were followed up in the outpatient clinic with receiving medication corresponding to their comorbidities.

### Statistical method

Before comparing the variables between the two groups, we first conducted a normality test to ensure that each group exhibited normal distribution. A total of 314 patients were enrolled in the study, and the Shapiro–Wilk test was used to verify normality. After confirming normality, we assessed homogeneity of variance using the Levene test. Once normality and homogeneity of variance were established, we proceeded with parametric tests. In this study, for baseline characteristics, descriptive statistical analysis is conducted using the t-test and chi-square test. The t-test is employed to compare the means of continuous variables between two groups, while the chi-square test is used to compare the proportions of categorical variables between different groups.

When comparing the time until the occurrence of an end point (event), Kaplan–Meier estimation and Cox proportional hazard ratio analysis are performed. Kaplan–Meier analysis estimates the survival time from the observed data, providing a visual representation of the survival or event-free probability over time. On the other hand, Cox-proportional hazard ratio analysis was used to assess the effect of multiple covariates on the hazard or event occurrence rate. The statistical program used for the analyses was SPSS version 23.0. Statistical significance was evaluated based on a p-value threshold of less than 0.05, indicating that any results with a p-value below this threshold were considered statistically significant.

### Ethical statement

This study was approved by the ethics committees of the appropriate institutional review board (IRB) of Asan Medical Center (IRB No.2022–0685). Informed consent was waived by the Institutional Review Board considering the retrospective nature of the study. All methods were carried out in accordance with relevant guidelines and regulations.

## Data Availability

The datasets used and/or analysed during the current study available from the corresponding author on reasonable request.

## Abbreviations

CRP: C-reactive protein
FLAIR: Fluid attenuated inversion recovery
HDL: High density lipoprotein
ICA: Internal carotid artery
LDL: Low density lipoprotein
PCA: Posterior cerebral artery
rCVR: Relative cerebral vascular reserve
MMD: Moyamoya disease
mRS: Rankin scale
RNF 213: Ring finger protein 213
SPECT: Single positron emission computed tomography
TFCA: Transfemoral cerebral angiography
TG: Triglyceride
WBC: White blood cell
